# Association of Rituximab Use With Adverse Events in Children, Adolescents, and Young Adults

**DOI:** 10.1001/jamanetworkopen.2020.36321

**Published:** 2021-02-03

**Authors:** Casey Lee McAtee, Joseph Lubega, Kristen Underbrink, Kristen Curry, Pavlos Msaouel, Martha Barrow, Eyal Muscal, Timothy Lotze, Poyyapakkam Srivaths, Lisa R. Forbes, Carl Allen, M. Brooke Bernhardt

**Affiliations:** 1 Section of Hematology-Oncology, Department of Pediatrics, Baylor College of Medicine, Houston, Texas; 2 Department of Pharmacy, Texas Children’s Hospital, Houston; 3 Department of Genitourinary Medical Oncology, Division of Cancer Medicine, University of Texas MD Anderson Cancer Center, Houston

## Abstract

**Importance:**

Rituximab is among the most frequently used immunotherapies in pediatrics. Few studies have reported long-term adverse events associated with its use for children.

**Objective:**

To describe the use of rituximab and to assess whether its use is associated with short- or long-term adverse events, infections, or time to immune reconstitution in a diverse group of young people.

**Design, Setting, and Participants:**

This retrospective cohort study included 468 patients aged younger than 21 years who received rituximab for diverse indications between October 1, 2010, and December 31, 2017, at Texas Children’s Hospital, a large pediatric referral hospital. Patterns of adverse events, infections, and immune recovery are described. Data analyses were conducted from December 2019 to June 2020.

**Exposure:**

One or more doses of rituximab.

**Main Outcomes and Measures:**

Adverse drug events (eg, anaphylaxis), incidence of mild and severe infections, and time to recovery of B lymphocyte subset counts and immunoglobulin levels. Survival models and logistic regression analyses and were used to identify associated risk factors of infectious and noninfectious adverse drug events.

**Results:**

We identified 468 patients receiving at least 1 dose of rituximab. The total follow-up time was 11 713 person-months. Of the 468 patients, 293 (62.6%) were female, the median (interquartile range) age at receipt of dose was 14.3 (9.9-16.8) years, and 209 (44.7%) were self-reported White Hispanic. Adverse events associated with rituximab infusion occurred in 72 patients (15.4%), and anaphylaxis occurred in 17 patients (3.6%). Long-term adverse events, such as prolonged neutropenia and leukoencephalopathy, were absent. Infections occurred in 224 patients (47.9%); 84 patients (17.9%) had severe infections, and 3 patients (0.6%) had lethal infections. Concurrent use of intravenous chemotherapy, treatment of systemic lupus erythematosus, neutropenia, and use of intravenous immunoglobulin were associated with increased risk of infection. Among 135 patients (28.8%) followed up to B cell count recovery, CD19^+^ or CD20^+^ cell numbers normalized in a median of 9.0 months (interquartile range, 5.9-14.4 months) following rituximab use; 48 of 95 patients (51%) evaluated beyond a year had low-for-age B cell counts. Recovery of CD27^+^ memory B cell number occurred in a median of 15.7 months (interquartile range, 6.0-22.7 months). Among patients with normal baseline values, low immunoglobulin G (IgG) levels developed in 67 of 289 patients (23.2%) and low IgM levels in 118 of 255 patients (40.8%); of these patients evaluated beyond 12 months from rituximab, 16 of 117 (13.7%) had persistently low IgG and 37 (33.9%) of 109 had persistently low IgM.

**Conclusions and Relevance:**

Rituximab is well tolerated among young people and is associated with few serious adverse events, but infections are common, corresponding to a prolonged period of B cell count recovery often lasting for longer than a year. Further examination of strategies to prevent infections following rituximab should be pursued.

## Key Points

**Question:**

Is the use of rituximab for young people associated with short- or long-term adverse events?

**Findings:**

This cohort study identified 468 patients younger than 21 years receiving rituximab for more than 25 indications, among whom infectious and noninfectious adverse events were common. The majority of these events were mild, but a small population experienced prolonged immune suppression and severe infections following even single courses of rituximab.

**Meaning:**

Findings suggest that rituximab appears to be well tolerated among young people, but the observed frequent infections and prolonged recovery of B lymphocyte numbers highlight the need for better strategies to mitigate infection risk in this population.

## Teaser

This cohort study conducted at a large pediatric referral hospital assesses whether the use of rituximab for many diverse indications is associated with short- or long-term adverse events among patients younger than 21 years.

## Introduction

Targeted immunotherapy is a mainstay for treatment of malignant neoplasms, autoimmune disease, and other disorders of immune dysregulation.^1,2,3^ Although harnessing the immune system has changed the treatment landscape for many diseases, these therapies may bring with them risks and adverse secondary events.

Rituximab is a chimeric human/murine monoclonal antibody against CD20 that depletes B cells through immune-mediated cytotoxicity and induction of apoptosis.^4,5^ It induces prolonged B-cell count suppression and diminishes circulating antibodies.^2,6,7^ B lymphocyte numbers typically recover within 6 to 12 months, but few studies have described long-term immune reconstitution in children following treatment with rituximab, particularly across its broad range of indications.^2,6^

The adverse events associated with rituximab include infusion reactions, anaphylaxis, and infections.^3,8,9^ Rare but serious events—described primarily in adults—include progressive multifocal encephalopathy, prolonged neutropenia, and fatal viral reactivation.^10,11,12,13^ Data regarding risk factors associated with adverse drug reactions, infections, and prolonged immune recovery are limited, being derived primarily from studies of adults or small, single-disease cohorts in children.^3,5,9,14^

Adverse events associated with rituximab use are difficult to predict and, in turn, cause difficult treatment quandaries. The objective of the present study was to investigate issues relevant to the pediatrician or subspecialist caring for patients following courses of rituximab. First, we describe short- and long-term adverse events associated with rituximab use. We further investigate infections and their associated risk factors. Finally, we describe patterns of B-cell recovery and immunoglobulin recovery.

## Methods

### Study Design

We conducted a retrospective cohort study of patients younger than 21 years receiving rituximab at Texas Children’s Hospital between October 1, 2010, and December 31, 2017. Person-time in the study spanned between patients’ first rituximab dose and their last follow-up date or the end of the study period, whichever occurred first. To avoid confounding by other major causes of prolonged lymphodepletion and infection, we excluded patients receiving rituximab for hematopoietic stem cell or solid organ transplant, those receiving other lymphodepleting therapy (eg, alemtuzumab), and those with primary immunodeficiencies. Those receiving rituximab prior to the study period—identified via electronic search of the medical record—were excluded. This study followed the Strengthening the Reporting of Observational Studies in Epidemiology (STROBE) reporting guideline. Owing to its retrospective nature, the study was approved with a waiver for the requirement of obtaining informed consent by the institutional review board of Baylor College of Medicine in Houston, Texas. No one received compensation or was offered any incentive for participating in this study.

### Data Collection

We extracted data from the electronic medical record, including demographic reports, patient problem lists, microbiology results, laboratory data (including flow cytometry results for peripheral blood lymphocyte subsets), and allergy lists. Race and ethnicity were self-reported. The record of each patient was scrutinized for keywords developed by the study team to ensure that relevant events were captured. Notes documenting rituximab infusions were manually reviewed. Because we studied patients in routine clinical practice, adverse events were captured only when they reached medical attention.

Infections and adverse drug events were classified and graded according to the Common Terminology Criteria for Adverse Events (CTCAE) version 5.0.^15^ Events that are CTCAE grade 3 or higher require intravenous medications or hospitalization; they are designated “severe” in this study. Anaphylaxis was defined per the National Institute of Allergy and Infectious Diseases and Food Allergy and Anaphylaxis Network.^16^ All anaphylactic events were confirmed by the investigators.

### Statistical Analysis

#### Modeling Adverse Drug Events

To evaluate risk of adverse drug events following a single course of rituximab, we used multivariable Cox proportional hazards models with stepwise selection.^17^ A rituximab “course” was a group of at least 1 dose with less than 1 month between doses. Patients were right censored at study end, last follow-up, or 2 years beyond rituximab use, whichever occurred first. We defined the period at risk as 2 years from a course given in which B cells generally recover within 24 months following rituximab use.^2,6^ Adverse events occurring outside of this 2-year window were reported but were not included in the survival models.

Risks of the following outcomes were assessed independently: incident infections, incident hypogammaglobulinemia, incident neutropenia (≤1000/μL [to convert neutrophils to ×10^9^/L, multiply by 0.001]), serum sickness, multifocal leukoencephalopathy, pneumonitis, and secondary malignant neoplasm. To model risk of recurrent infections, a gamma-distributed shared frailty term was added to the model to account for clustering by patient.^18,19^ Documenting neutropenia or hypogammaglobulinemia relied on data captured in routine practice; otherwise, data were complete. To model risk of reactions associated with infusion—potentially recurrent events occurring during or immediately after infusions—we used multivariable logistic regression models with mixed-effects modeling to account for clustering by patient.^20^

#### Model Risk Factors

The risk factors assessed were age, sex, race/ethnicity, number of previous rituximab doses, oncology diagnosis, lupus diagnosis, concurrent oral immunosuppression (mycophenolate mofetil or corticosteroid), and concurrent intravenous immunoglobulin (IVIG). For infections, concurrent *Pneumocystis jiroveci* prophylaxis and neutropenia (as a dichotomous time-varying covariate) were added. For reactions associated with infusion, preinfusion prophylaxis (hydrocortisone, diphenhydramine, or acetaminophen) was added.

Neutropenia was assessed a median of twice monthly (interquartile range [IQR], 1-4 evaluations per month) during the follow-up period. Sparsity of immunoglobulin level and B-cell count data at approximate times of infections prohibited their inclusion as risk factors in the infection model; otherwise, data were complete. All variables included in the adjusted models met the assumption of proportional hazards using Schoenfeld residuals.^21,22^

As a post hoc analysis, we used a univariable Cox proportional hazards model as described above to determine the association between IVIG use and infections in the year prior to patients’ first dose of IVIG. Time at risk began either 365 days prior to the first dose of rituximab or the patient’s first clinical encounter, whichever occurred later. Time at risk ended on the day of dose 1 of rituximab.

All tests were 2-sided with α = .05. All analyses were performed from December 2019 to June 2020 in R with use of the Survival and lme4 packages.^22,23,24^

## Results

We identified 468 patients receiving rituximab during the study period (Table 1). Of the 468 patients, 293 (62.6%) were female, the median age at dose was 14.3 years (IQR, 9.9-16.8 years), and 209 (44.7%) were self-reported White Hispanic. An additional 276 patients were excluded, most commonly for lymphodepletion prior to stem cell transplant (115 [41.7%]) or solid organ transplant (108 [39.1%]) (eTable 1 in the Supplement). The total follow-up time was 11 713 person-months, with a median follow-up of 21.2 months (IQR, 9.0-37.7 months). The most common indications for rituximab were systemic lupus erythematosus (124 [26.5%]), nephrotic syndrome (61 [13.0%]), autoimmune encephalitis (42 [9.0%]), and lymphoma (30 [6.4%]). Patients most often received a single dose (63 [13.5%]) or a single multidose course of rituximab (253 [54.3%]) with a median of 2 doses per course (range, 2-9 doses). Preinfusion prophylaxis with corticosteroids, acetaminophen, or diphenhydramine was used prior to 1371 of 1714 total doses (80.0%).

**Table 1.** Demographic and Clinical Characteristics of 468 Patients Receiving Rituximab

Demographic characteristic	Patients, No. (%)
Female sex	293 (62.6)
Age at dose, median (interquartile range), y	14.3 (9.9-16.8)
Ethnicity	
White Hispanic	209 (44.7)
Non-Hispanic	
White	121 (25.9)
Black	99 (21.2)
Asian	23 (4.9)
Other	16 (3.4)
**Rituximab courses**	
Indication for rituximab	
Systemic lupus erythematosus	124 (26.5)
Nephrotic syndrome	61 (13.0)
Autoimmune encephalitis	42 (9.0)
Lymphoma	30 (6.4)
Immune thrombocytopenic purpura	26 (5.6)
Multiple sclerosis	23 (4.9)
Other	162 (34.6)
Dosing schedule	
Single	63 (13.5)
Single multidose course^a^	253 (54.3)
Doses per course, median (range)	2 (2-9)
Multiple courses	152 (32.5)
Concurrent medications	
Corticosteroids	356 (76.1)
Mycophenolate mofetil	126 (26.9)
Tacrolimus	22 (4.7)
Pneumocystis *jiroveci* prophylaxis^b^	102 (21.8)

^a^ Two or more doses with less than 1 month between doses, and no subsequent doses.

^b^ Trimethoprim-sulfamethoxazole, pentamidine, atovaquone, and/or dapsone.

### Immediate and Long-term Noninfectious Adverse Drug Events

Noninfectious adverse drug events were common (Table 2). Of 468 patients, 17 (3.6%) experienced anaphylaxis, most often after the first (n = 9) or second (n = 3) dose, but it occurred as late as the eighth dose following previously uneventful infusions. Other reactions associated with infusion occurred in 72 patients (15.4%), typically after the first dose (52 patients [72.2%]). Following these reactions, most patients (63 [87.5%]) were able to complete the infusion and were given subsequent doses without a recurrence. Transient hypotension, respiratory distress, or rigors resolving after pausing the infusion or giving oral antihistamines occurred most commonly.

**Table 2.** Adverse Drug Events and Infections Among Patients Receiving Rituximab

Adverse drug event	Patients, No. (%)
Anaphylaxis	17 (3.6)
Signs/symptoms	
Respiratory compromise	10 (2.1)
Gastrointestinal tract symptoms	8 (1.7)
Skin or mucosal involvement	7 (1.5)
Hypotension	4 (0.8)
Received epinephrine	6 (1.3)
Infusion reaction^a^	72 (15.4)
Signs/symptoms	
Hypotension	16 (3.4)
Cough, wheezing, or dyspnea	14 (3.0)
Rigors	14 (3.0)
Hives, rash, or generalized pruritus	12 (2.6)
Chest or throat tightness	10 (2.1)
Headache	7 (1.5)
Fever	6 (1.3)
Nausea, vomiting, or abdominal pain	6 (1.3)
Hypertension	5 (1.1)
CTCAE grade ≥3 infusion reaction^b^	7 (1.5)
Infusion terminated	9 (1.9)
Neutropenia^c^	3 (4.2)
Hypogammaglobulinemia^b^	
IgG	67 (23.2)
IgM	104 (40.8)
Serum sickness	3 (0.6)
Infection	
Any severity	224 (47.9)
Severe^d^	84 (17.9)
Requiring ICU	17 (3.6)
Death	3 (0.6)

Abbreviations: CTCAE, Common Terminology Criteria for Adverse Events; ICU, intensive care unit; IgG, immunoglobulin G; IgM, immunoglobulin M.

^a^ Infusion reactions excluding anaphylaxis.

^b^ New-onset hypogammaglobulinemia only (normal baseline).

^c^ Limited to patients not taking immunosuppressive medications known to cause neutropenia (n = 72).

^d^ Infections associated with CTCAE grade 3 events typically requiring intervention with intravenous medications, hospitalization, or both.

Moderate neutropenia (<1000/μL) occurred in 95 patients (20.3%), and severe neutropenia (<500/μL) occurred in 66 patients (14.1%). To isolate the association between rituximab and neutropenia, we analyzed 72 patients who were not taking other medications known to cause neutropenia. In this subcohort, moderate neutropenia occurred in 3 patients; it lasted longer than a month in a single patient and resolved within 2 months.

Owing to the low incidence of other noninfectious adverse events (21 [4.5%]), only risk factors of neutropenia, hypogammaglobulinemia, and infusion reactions were analyzed in the regression analysis. Increasing the number of rituximab doses was associated with increased risk of neutropenia (hazard ratio [HR], 1.64; 95% CI, 1.33-2.04) in the adjusted model (Table 3). Patients receiving IVIG were more likely to have hypogammaglobulinemia (HR, 2.56; 95% CI, 1.38-4.74). The odds of infusion reactions decreased with successive doses (odds ratio, 0.30; 95% CI, 0.20-0.45). Preinfusion prophylaxis did not decrease the odds of infusion reactions.

**Table 3.** Cox Proportional Hazards Model of Risk Factors for Neutropenia or Hypogammaglobulinemia Following a Single Course of Rituximab

Risk factor	Unadjusted model		Adjusted model	
	HR (95% CI)	*P* value	HR (95% CI)	*P* value
Neutropenia (<1000/μL)^a^				
Year of age (increasing)	0.94 (0.89-0.99)	.03	0.94 (0.89-1.0)	.05
Hispanic race/ethnicity	3.40 (0.45-25.50)	.23	ND	ND
Female sex	0.72 (0.40-1.31)	.28	ND	ND
Oncology diagnosis	13.86 (6.98-27.54)	<.001	6.05 (2.53-14.46)	<.001
No. of doses (increasing)	1.93 (1.63-2.29)	<.001	1.64 (1.33-2.04)	<.001
Concurrent medication				
Mycophenolate mofetil	1.13 (0.60-2.10)	.70	2.35 (1.12-4.93)	.03
Corticosteroid	1.49 (0.69-3.22)	.31	ND	ND
Hypogammaglobulinemia (IgG)^b^				
Year of age (increasing)	0.97 (0.91-1.03)	.27	ND	ND
Hispanic race/ethnicity	0.77 (0.41-1.44)	.41	ND	ND
Female sex	0.88 (0.45-1.71)	.70	ND	ND
Oncology diagnosis	2.97 (1.16-7.57)	.02	1.88 (0.59-5.99)	.29
No. of doses (increasing)	1.26 (1.03-1.55)	.03	1.23 (0.95-1.60)	.12
Concurrent medication				
Mycophenolate mofetil	0.94 (0.51-1.73)	.84	ND	ND
Corticosteroid	2.21 (0.68-7.14)	.19	ND	ND
IVIG	2.39 (1.30-4.38)	.005	2.56 (1.38-4.74)	.003

Abbreviations: HR, hazard ratio; IgG, immunoglobulin G; IVIG, intravenous immunoglobulin; ND, not determined.

SI conversion factors: To convert neutrophils to ×10^9^/L, multiply by 0.001.

^a^ For neutropenia, data represent 307 patients receiving a single course of rituximab with neutrophil count data available.

^b^ For hypogammaglobulinemia, these data similarly represent 252 patients.

A single child developed cancer following rituximab use; Hodgkin lymphoma (Epstein-Barr virus negative) was diagnosed 14 months after treatment with rituximab for immunoglobulin G4 (IgG4)–related sclerosing disease. There were no cases of progressive multifocal leukoencephalopathy or pneumonitis.

### Infections

Infections were most common in the 6 months following rituximab use, but their incidence continued to trend downward for 2 years following doses (χ^2^ trend test, *P* < .001). Severe infections were most common in the first month (Figure, A).

**Figure.**
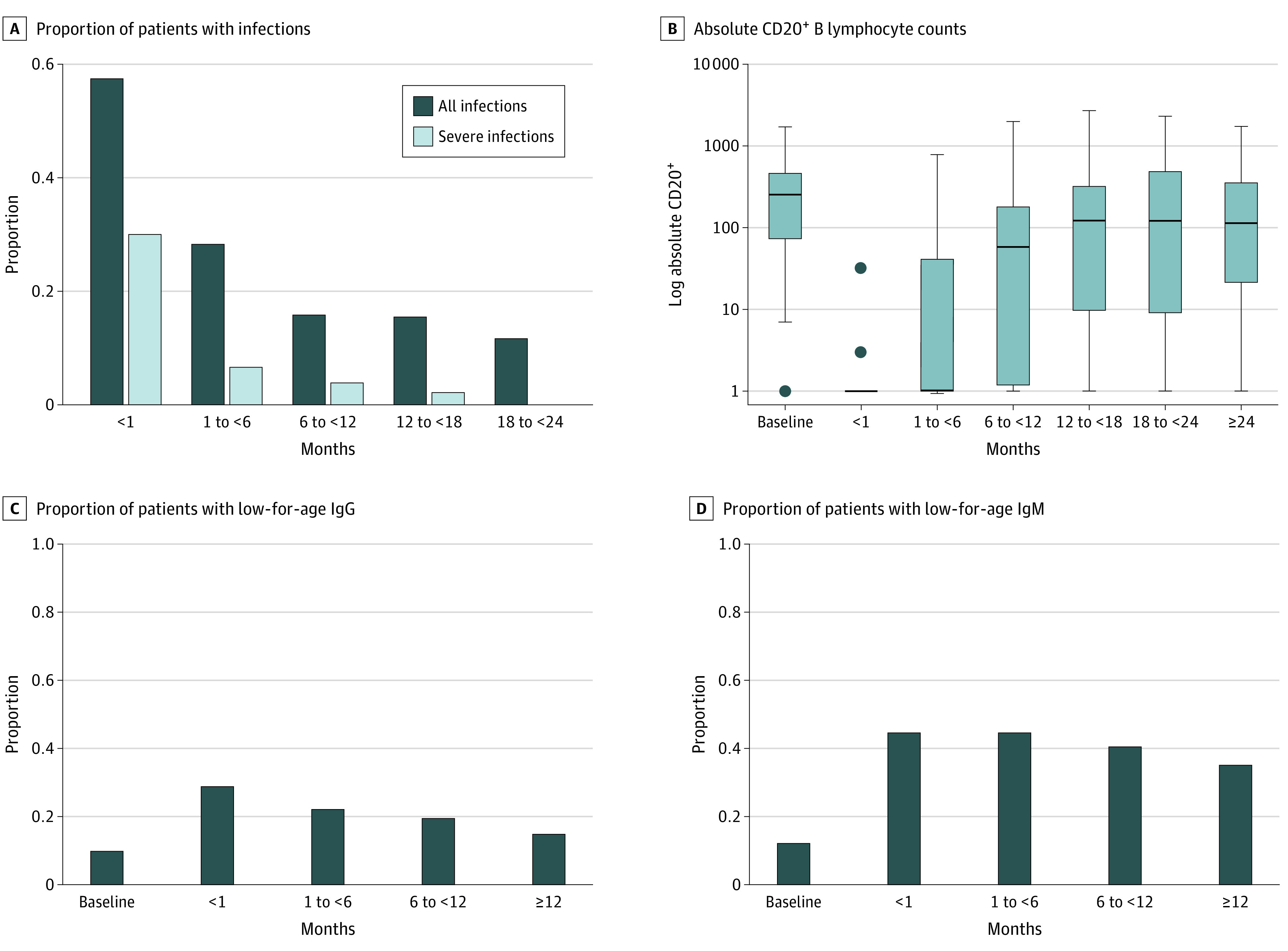
B Lymphocyte Recovery, Hypogammaglobulinemia, and Infections at Intervals Following Single Courses of Rituximab A, Denominator of patients under follow-up at less than 1 month, 316; 1 month to less than 6 months, 294; 6 months to less than 12 months, 240; 12 months to less than 18 months, 186; and 18 months to less than 24 months, 148. B, Box and whisker plot with data log_10_ transformed for graphical depiction. A constant of 1 was added to all absolute CD20^+^ values prior to log_10_ transformation to avoid noninfinite values where absolute CD20^+^ = 0. Number of tests obtained at baseline, 92; less than 1 month, 104; 1 month to less than 6 months, 222; 6 months to less than 12 months, 146; 12 months to less than 18 months, 60; 18 months to less than 24 months, 34; and 24 months or longer, 44. The lower and upper ends of the whisker represent minimum and maximum counts for each interval. The lower border of the box represents the 25th percentile; the upper border represents the 75th percentile; the horizontal band, the median; and the solid dots, outliers. C, Denominator of patients under follow-up at baseline, 326; less than 1 month, 236, 1 month to less than 6 months, 294; 6 months to less than 12 months, 210; and 12 months or longer, 142. D, Denominator of patients under follow-up at baseline, 292; less than 1 month, 191; 1 month to less than 6 months, 267; 6 months to less than 12 months, 198; and 12 months or longer, 137.

Within 2 years of receiving rituximab, infections occurred in 224 patients (47.9%); 84 patients (17.9%) had severe infections, and 3 patients (0.6%) had lethal infections.

Infections were more common among patients treated for oncologic vs nononcologic indications (HR, 3.50; 95% CI, 1.37-9.00); therefore, we report incidence separately for each group. Infections occurred in 198 of 428 nononcology patients (46.3%) and 26 of 40 oncology patients (65.0%) within 2 years of receiving rituximab. Severe infections (CTCAE grade ≥3) occurred in 67 nononcology patients (15.7%) and 17 oncology patients (42.5%). Severe infections were high immediately after a dose, with 52 of 129 (40.3%) occurring within a month. Multiple infections were common, occurring in 98 nononcology patients (22.9%; median number of infections, 3 [IQR, 2-4]) and 20 oncology patients (50.0%; median number of infections, 3 [IQR, 2-5]). In total, the incidence density of infections in the cohort was 64 infections per 100 patient-years; for severe infections, it was 13 per 100 patient-years.

The most common of 481 infections were upper respiratory tract infections (60 [12.5%]), lower respiratory tract infections (57 [11.9%]), urinary tract infections (50 [10.4%]), and skin or soft tissue infections (43 [8.9%]). The most common of 129 severe infections were lower respiratory tract infections (28 [21.7%]), bacteremia (14 [10.9%]), acute gastroenteritis (11 [8.5%]), and urinary tract infections (10 [7.8%]). Seventeen patients (3.6%) required intensive care, and 3 (0.6%) died. All deaths were among nononcology patients.

Confirmed viral infections occurred in 40 patients (8.5%), most commonly influenza (n = 26) and herpes simplex virus (n = 9). Five patients with herpes simplex infections required hospitalization. Epstein-Barr virus was detected in 3 patients, and cytomegalovirus in 1 patient; however, surveillance of these viruses was not routine. There were no cases of *Pneumocystis jiroveci* pneumonia.

Along with cancer diagnosis, neutropenia (HR, 2.40; 95% CI, 1.17-4.91), treatment of systemic lupus erythematosus (HR, 1.98; 95% CI, 1.18-3.32), and concurrent use of IVIG (HR, 2.23; 95% CI, 1.41-3.54) were associated with increased risk of infections in the adjusted model (Table 4). Owing to their high collinearity, nearly identical findings were observed when lupus diagnosis was replaced with concurrent use of mycophenolate mofetil (ie, most patients with lupus were taking mycophenolate, and vice versa). These risk factors were similarly associated with severe infections (eTable 2 in the Supplement).

**Table 4.** Cox Proportional Hazards Model of Risk Factors for Infections Among 315 Patients Following a Single Course of Rituximab

Risk factor	Unadjusted model		Adjusted model	
	HR (95% CI)	*P* value	HR (95% CI)	*P* value
Demographic characteristic				
Female sex	0.95 (0.67-1.35)	.79	ND	ND
Hispanic race/ethnicity	1.24 (0.89-1.73)	.20	ND	ND
Year of age (increasing)	0.97 (0.94-1.00)	.06	ND	ND
Diagnosis and rituximab dosing^a^				
No. of doses (per dose)	1.15 (1.03-1.29)	.01	1.00 (0.82-1.24)	.96
Oncologic diagnosis	2.36 (1.46-3.80)	<.001	3.50 (1.37-9.00)	.009
Lupus erythematosus	1.13 (0.78-1.64)	.51	1.98 (1.18-3.32)	.009
Moderate neutropenia^b^	3.71 (1.90-7.26)	<.001	2.40 (1.17-4.91)	.02
Severe neutropenia^b^	5.17 (2.51-10.66)	<.001	ND	ND
Concurrent medication				
Intravenous immunoglobulin	1.65 (1.19-2.30)	.003	2.23 (1.41-3.54)	<.001
PJP prophylaxis^c^	1.41 (0.96-2.07)	.08	ND	ND
Corticosteroids	1.19 (0.84-0.79)	.40	ND	ND
Mycophenolate mofetil	1.33 (0.93-1.90)	.12	ND	ND

Abbreviations: HR, hazard ratio; ND, not determined; PJP, *Pneumocystis jiroveci*.

SI conversion factors: To convert neutrophils to ×10^9^/L, multiply by 0.001.

^a^ Only diseases with more than 50 participants tested as risk factors.

^b^ Moderate (<1000/μL) and severe (<500/μL) neutropenia were time-varying covariates. Owing to collinearity, only moderate neutropenia was included in the multivariable model.

^c^ *Pneumocystis jiroveci* pneumonia prophylaxis included trimethoprim-sulfamethoxazole, pentamidine, atovaquone, and dapsone.

Of patients with infections who received IVIG following rituximab, 63 (64.3%) were given at least 1 dose of IVIG prior to their first infection. As well as being at higher risk of infection in the period after rituximab, those receiving IVIG also had a higher risk of infection in the year prior to their first dose of rituximab (HR, 3.67; 95% CI, 2.19-6.12).

### Immune Reconstitution

Clinicians obtained lymphocyte subsets for a median of 6.3 months (IQR, 2.8-13.3 months) after courses of rituximab. Peripheral absolute B-cell counts decreased rapidly following rituximab use and generally recovered within 24 months following the last dose (Figure, B). Of 104 evaluations of CD20 lymphocyte counts within 1 month of rituximab, 100% were low-for-age and only 2 were above 0.

Of 468 patients, 135 (28.8%) had B lymphocyte counts followed up to normalization of either CD19^+^ or CD20^+^ cell counts after a course of rituximab (laboratory evaluations variably reported CD19^+^, CD20^+^, or both populations). Patients achieved normalization of either population in a median of 9.0 months (IQR, 5.9-14.4 months), up to a maximum of 52.8 months. Of 95 patients evaluated beyond a year, 48 (51%) had persistently low levels. Recovery of CD19^+^CD27^+^ memory B cell counts occurred in a median of 15.7 months (IQR, 6.0-22.7 months).

Patients with lupus followed up to normalization of CD20^+^ or CD19^+^ lymphocyte counts after a single course of rituximab required a median of 15.3 months (IQR, 12.5-27.9 months) to normalize compared with 9.4 months (IQR, 6.2-13.8 months) for other patients (Wilcoxon rank sum *P* = .002). Although patients with lupus were evaluated at similar intervals and durations as the entire cohort, the potential for ascertainment bias influencing those receiving follow-up evaluations must be considered here.

Immunoglobulin levels were available for 358 patients (76.5%). Hypogammaglobulinemia was common throughout the first year following rituximab use (Figure, C and D). Clinicians followed immunoglobulin levels for a median of 6.1 months (IQR, 0.9-13.8 months) after rituximab courses. Of 289 patients with normal baseline immunoglobulin levels, 67 (23.2%) were documented to have low IgG levels after rituximab courses, and 104 of 255 patients (40.8%) were documented to have low IgM levels after rituximab courses. Among those patients, 51 (76.1%) were followed up to normalization of IgG, and 64 (61.5%) were followed up to normalization of IgM. Of patients with normal baseline immunoglobulin levels evaluated later than 12 months from rituximab, 16 of 117 (13.7%) had persistently low IgG and 37 of 109 (33.9%) had persistently low IgM.

## Discussion

We investigated the immediate and long-term adverse events associated with rituximab therapy in a diverse cohort of 468 children, adolescents, and young adults treated for more than 25 disorders. The heterogeneity of patient age, race/ethnicity, and diagnoses allowed for an assessment of the practical challenges in the care of young people receiving rituximab. Trends in these data may inform clinical care and the investigations surrounding risk factors of adverse drug events, as well as the appropriate timing of laboratory evaluation and vaccination for children.

The reactions associated with rituximab infusion in the present study were uncommon and rarely severe; more than three-quarters of patients with infusion reactions were safely given subsequent doses. Clinical trials involving rituximab report infusion reactions in 50% to 90% of patients, but these reactions were mild in most cases.^3,25,26,27^ Outside of clinical trials, infusion reactions are documented at much lower rates—reflective of their low acuity.^28,29^ The incidence of life-threatening events, such as anaphylaxis, is 1% to 4% across studies.^2,26,29,30^ As in our cohort, reactions most often occur following the first dose, but reactions following later doses are possible.^3,26^

Severe events, such as leukoencephalopathy and prolonged neutropenia, were absent in the present study, suggesting that if they do occur in young people, they are extremely rare.^2,12,13^ Lethal viral reactivation was also absent in the present study, but rare and severe herpesvirus infections in the cohort highlight the importance of expectant management of viral infections in these children. The single incident case of cancer occurred in a patient with IgG4-related disease, a disease unassociated with cancer.^31^ Whether rituximab increases risk of cancer remains uncertain.^32,33^

The reported prevalence of infection following rituximab use is highly variable. The figures range from 10% to 70%, reflecting the heterogeneity of the populations and the study designs implemented.^2,27,34^ When infections are narrowed to the defined criterion of requiring hospitalization, the incidence becomes more consistent, ranging from 5 to 10 per 100 person-years, similar to the present cohort.^28,29,35^

Infections were most common in the first 6 months following rituximab use in the present study, and incidence trended downward as immune recovery occurred during 2 years (Figure). Severe infections were particularly high immediately after a dose—40.3% of 129 occurred within a month—highlighting this period as one in which infection-mitigating strategies should be focused.

Patients receiving IVIG were more likely to have infections, even after controlling for other causes of infection, such as neutropenia and concurrent immunosuppression (Table 4). This unexpected finding was previously described in adults treated with rituximab and IVIG.^14^ We emphasize that this adverse “effect” associated with IVIG use is most consistent with reverse causality, the phenomenon whereby patients at higher risk for infection were given IVIG, thus creating the association. That patients receiving IVIG were more likely to have documented hypogammaglobulinemia supports this hypothesis. Furthermore, although most patients received IVIG prior to their first infection, infection incidence was also higher in the IVIG group during the year preceding rituximab, suggesting that unmeasured confounders produced the association between IVIG and increased risk of infection.

To date, no randomized clinical trials have reported the efficacy of IVIG following rituximab use to prevent infections. Data supporting the use of IVIG following cytotoxic chemotherapy are mixed; IVIG may be associated with decreased risk of infection in patients with leukemia or lymphoma, but this decreased risk is insignificant following stem cell transplant.^36,37,38,39^

We should consider the possibility that IVIG does not consistently prevent infections in patients receiving rituximab. First, because IVIG contains only trace amounts of IgM—the deficiency of which is linked to increased infection risk—it will not correct the pronged deficiency of IgM commonly induced by rituximab.^40,41^ Second, because IVIG has a half-life of approximately 30 days, patients may require IVIG for longer courses to experience a benefit.^42^ Third, replenishment of IgG may be insufficient to overcome other major causes of infection, such as neutropenia.^38^ Ultimately, a randomized clinical trial studying the prophylactic use of IVIG—a medication with its own adverse drug events—should be conducted among children after rituximab use.

The evaluation of B-cell count recovery following rituximab is not standardized. Evaluation within a month of a dose to confirm lymphodepletion is unnecessary in most cases; of 104 CD20^+^ evaluations, all had low-for-age values, and only 2 had a value greater than 0 (Figure, B). Similar to other studies, B cell counts in young people evaluated in the present study usually recovered within a year, but recovery was often delayed.^2,7,27,43^ Approximately half of the patients evaluated beyond a year had low-for-age B cell counts, suggesting that their prolonged suppression is common in children.

This cohort underscores the substantial population of children with diminished numbers of B lymphocytes for more than a year after receiving single courses of rituximab. Patients treated for lupus, in particular, required nearly a year and a half for recovery. Although the possibility of ascertainment and selection bias must be considered when interpreting these data, the increased risk of infection in patients with lupus described here highlights the importance of further study of infections and immune reconstitution in this population.

Finally, CD19^+^CD27^+^ memory B cell numbers were especially slow to recover (median, 15.7 months). Memory B cells are required for adequate vaccine response—responses that are attenuated in adults following rituximab use.^44,45,46^ Based on these data and the highly variable, prolonged time to recovery of memory B cell numbers in children, it may be necessary for clinicians to either document the return of these cells prior to vaccination or confirm adequate vaccine antibody titers following routine childhood vaccination.

### Strengths and Limitations

To our knowledge, this is the largest study to describe adverse drug events among children receiving rituximab across the broad spectrum of its use. A retrospective cohort design was necessary to study its use in the settings of routine clinical practice; thus, the study has the intrinsic weaknesses of retrospective cohorts, particularly the risk of misclassification bias due to incompleteness or miscoding in the medical record. Furthermore, subclinical events, particularly asymptomatic viral reactivations, were unlikely to be captured; however, these events are likely of limited clinical relevance. To reduce these risks, we used a multifaceted approach of searching the medical record.

Although these data showed long-term trends in immune reconstitution in a large group of children, adolescents, and young adults after rituximab use, the retrospective design risks ascertainment bias vis-à-vis unmeasurable influences on likelihood of laboratory evaluation. Furthermore, the sparseness of immunoglobulin and lymphocyte subpopulation data at times of infection precluded a more precise investigation of the associations between rituximab, hypogammaglobulinemia, and infection—thus, this should be studied prospectively alongside variables identified in this study as associated with infection risk.

## Conclusions

Rituximab is well tolerated in children, but infections after its use are common—corresponding to a prolonged period of depressed B cell numbers and immunoglobulin levels following even single courses of rituximab. Strategies to reduce infections following rituximab use should be studied prospectively, particularly the use of IVIG. Finally, the prolonged recovery of memory B cell numbers following rituximab use should be considered because it is associated with routine childhood vaccinations.

## Notes

Supplement. **eTable 1.** Indications for Study Exclusion Among 276 Patients **eTable 2.** Cox Proportional Hazards Model of Predictors of Severe Infections Following a Single Course of Rituximab
