# Supplementary material for: Association of Rituximab Use With Adverse Events in Children, Adolescents, and Young Adults
Source: JAMA Netw Open. 2021 Feb 3;4(2):e2036321. doi: 10.1001/jamanetworkopen.2020.36321 (PMC7859842; doi:10.1001/jamanetworkopen.2020.36321)
Supplement: Supplement. — eTable 1. Indications for Study Exclusion Among 276 Patients eTable 2. Cox Proportional Hazards Model of Predictors of Severe Infections Following a Single Course of Rituximab [file jamanetwopen-e2036321-s001.pdf]

Supplementary Online Content

McAtee CL, Lubega J, Underbrink K, et al. Association of rituximab use with adverse events in children, adolescents, and young adults. *JAMA Netw Open*. 2021;4(2):e2036321. doi:10.1001/jamanetworkopen.2020.36321

eTable 1. Indications for Study Exclusion Among 276 Patients

eTable 2. Cox Proportional Hazards Model of Predictors of Severe Infections Following a Single Course of Rituximab

This supplementary material has been provided by the authors to give readers additional information about their work.

© 2021 McAtee CL et al. *JAMA Network Open*.

eTable 1. Indications for Study Exclusion Among 276 Patients

Exclusion criterion	N (%)
Rituximab given as part of hematopoietic stem cell transplant regimen	115 (41.7)
Rituximab given as part of solid organ transplant regimen	108 (39.1)
No follow-up clinical data recorded	26 (9.4)
Primary immune deficiency	18 (6.5)
Concurrent therapy with alemtuzumab	4 (1.4)
Previous doses of rituximab	3 (1.1)
Aged ≥21 years	2 (0.7)

© 2021 McAtee CL et al. *JAMA Network Open*.

eTable 2. Cox Proportional Hazards Model of Predictors of Severe Infections Following a Single Course of Rituximab

Predictor	Unadjusted		Adjusted	
	HR (95% CI)	p-value	HR (95% CI)	p-value
Demographic				
Female sex	0.58 (0.31-1.08)	0.08	--	--
Hispanic ethnicity	1.925 (1.04-3.58)	0.04[d]	2.00 (0.90-4.44)	0.09
Year of age (increasing)	0.93 (0.87-0.98)	0.01[d]	0.93 (0.86-1.01)	0.10
Diagnosis and rituximab dosing[a]				
Number of doses (per dose)	1.34 (1.12-1.60)	**0.001**	0.99 (0.72-1.36)	0.93
Oncologic diagnosis	5.22 (2.60-10.47)	**<0.001**	6.25 (1.52-25.68)	**0.01**
Lupus erythematosus	1.12 (0.56-2.24)	0.75	2.63 (0.98-7.02)	0.05
Moderate neutropenia[b]	5.48 (2.30-13.05)	**<0.001**	2.84 (1.12-7.21)	**0.03**
Severe neutropenia[b]	5.44 (2.18-13.56)	**<0.001**	--	--
Concurrent medications				
Intravenous immunoglobulin	2.12 (1.15-3.92)	**0.02**	2.80 (1.24-6.34)	**0.01**
PJP prophylaxis[c]	1.54 (0.75-3.14)	0.24	--	--
Corticosteroids	0.87 (0.41-1.83)	0.71	--	--
Mycophenolate mofetil	1.29 (0.66-2.53)	0.46	--	--

Severe infections defined as Common Terminology Criteria for Adverse Events grade ≥3
[a]Only diseases with n>50 subjects tested as predictors
[b]Moderate (<1000 cells/µL) and severe (<500 cells/µL) neutropenia were time-varying covariates. Due to collinearity, only moderate neutropenia was included in the multivariable model.
[c]*Pneumocystis jiroveci* pneumonia prophylaxis included trimethoprim-sulfamethoxazole, pentamidine, atovaquone, and/or dapsone
[d]Failed proportional hazards assumption

© 2021 McAtee CL et al. *JAMA Network Open*.
